# Developments, Evolution, and Implications of National Diagnostic Criteria for COVID-19 in China

**DOI:** 10.3389/fmed.2020.00242

**Published:** 2020-05-15

**Authors:** Lin-Lu Ma, Bui-Hui Li, Ying-Hui Jin, Tong Deng, Xue-Qun Ren, Xian-Tao Zeng

**Affiliations:** ^1^Center for Evidence-Based and Translational Medicine, Zhongnan Hospital of Wuhan University, Wuhan, China; ^2^Institute of Evidence-Based Medicine and Knowledge Translation, Henan University, Kaifeng, China

**Keywords:** COVID-19, SARS-CoV-2, 2019-nCoV, diagnosis, guideline

## Abstract

Recently WHO has characterized COVID-19 as a pandemic. Diagnosing the disease accurately and decreasing misdiagnoses and missed diagnoses is very important for management. Therefore, we have analyzed the seven versions of China's national guidelines to examine how the diagnostic criteria roadmap has developed and evolved, in order to share our experience worldwide. In this article, we present the developments from the first to seventh versions, involving changes of case classification, changes to “suspected case,” changes in “confirmed case,” changes in clinical classifications, changes in “severe case,” and unchanged criteria. We have also discussed the reasons and implications for these changes and are looking forward to providing suggestions for worldwide understanding and management of this pandemic. A nucleic acid test is currently accepted as the gold standard method to confirm diagnosis. In addition, imaging examination and epidemiological history should also be considered as auxiliary diagnosis methods.

## Introduction

Since December 2019, a new disease caused by 2019 novel coronavirus (2019-nCoV) has resulted in a worldwide outbreak (1–3). The disease has been named coronavirus disease 2019 (COVID-19) and the virus has been named severe acute respiratory syndrome coronavirus 2 (SARS-CoV-2) (4). It is vital, both for individuals and governments, to have accurate diagnostic methods for this disease and to minimize misdiagnosis and missed diagnoses in order to facilitate prevention and treatment. Because COVID-19 is a new disease, our awareness and knowledge are gradually increasing based on ongoing research findings and clinical practice experience; hence, the diagnostic criteria are also evolving. The outbreak has continued to increase worldwide and on 11 March 2020, WHO characterized COVID-19 as a pandemic (5). Therefore, we aim to share our experience with the rest of the world based on an analysis of the evolving changes in the diagnostic criteria incorporated in the different versions of China's national guidelines for COVID-19.

## Methods

### Data Collection

We searched for all versions of the *Diagnosis and Treatment Guidelines for COVID-19*, which have been issued by the National Health Committee of the People's Republic of China (http://www.nhc.gov.cn/) up to 5 March 2020. The first to seventh versions were included and the data necessary to estimate the changes in diagnosis of COVID-19 were extracted. Two authors independently reviewed the full text. Discrepancies were resolved by discussion and consensus. Besides extracting all the context of diagnostic criteria, the issued data, clinical classification, and definition of severe disease were also collected.

### Statistical Analysis

All changes have been compared and presented in a separated table for suspected cases, confirmed cases, and severe cases. Other changed and unchanged items have also been described. All analyses used Microsoft Excel and PowerPoint 2019.

## Results

The first version was issued on 16 January 2020, and the seventh version on 3 March 2020. Table 1 presents the changes in these seven guidelines.

**Table 1:** The changes of diagnostic criteria for suspected case, confirmed case, and severe case in the seven national guidelines in China.

**Edition**	**Suspected case**	**Confirmed case**	**Severe case**
First^*^	•Epidemiological history: Travel history of visiting Wuhan within 2 weeks before onset; or direct or indirect contact with related markets in Wuhan. •Clinical manifestation: (1) fever; (2) having chest imaging features of COVID-19; (3) total white blood cell counts normal or decreased, or reduced lymphocyte count in the early onset stage; (4) condition fails to improve or shows progressive exacerbation after standardized antimicrobial therapy for 3 days.	The respiratory tract samples (sputum, oropharyngeal swabs) of observed cases for viral whole genome sequencing, showing high homogeneity to the known novel coronaviruses.	Not applicable.
Second	Must meet any of the two following items: •Epidemiological history: history of travel or residence in Wuhan within 2 weeks before onset; or a history of contact with patients with fever or respiratory symptoms from Wuhan in the in the last 14 days before symptom onset, or with a clustered of confirmed cases. •Clinical manifestation: (1) fever; (2) imaging features of COVID-19; (3) total white blood cell counts normal or decreased, or reduced lymphocyte count in the early onset stage.	The respiratory tract samples (sputum, oropharyngeal swabs, lower respiratory tract secretions) from suspected case for real-time PCR test for 2019-nCoV showing positive, or for viral whole genome sequencing showing high homogeneity to the known novel coronaviruses.	Any one of the following symptoms present: (1) Increased respiratory rate (≥30 breaths/minute), breathing difficulty or dyspnea, slightly cyanotic lips; or oxygen saturation during inhalation ≤ 95%, or the arterial partial pressure of oxygen (PaO2)/fraction of inspired oxygen (FiO2) ≤ 300 mmHg (1 mmHg = 0.133kPa); (2) Pulmonary imaging showing leafy lesions or progressive lesions > 50% in 48 h; (3) The rapid Sequential Organ Failure Assessment (qSOFA) score ≥ 2; (4) The CURB-65 score ≥ 1; (5) coalescent pneumothorax; (6) Other combined clinical conditions that necessitate hospitalization.
Third	Same as the second edition	Same as the second edition.	Meets any one of the following criteria: (1) Increased the respiratory rate (≥30 breaths/minute), dyspnea, lips slightly cyanosed; (2) The oxygen saturation during inhalation ≤ 93%; (3) The PaO2/ FiO2 ≤ 300 mmHg (1 mmHg = 0.133kPa); (4) Pulmonary imaging shows leafy lesions or progressive lesions > 50% in 48 h; (5) Other combined clinical conditions that necessitate hospitalization.
Fourth	Combination of any one feature of epidemiological history with two clinical manifestations to make a comprehensive analysis: •Epidemiological history: (1) a history of travel or residence in Wuhan city or other places where COVID-19 had spread in the last 14 days before symptom onset; (2) a history of contact with patients with fever or respiratory symptoms from Wuhan city or other places where COVID-19 had spread in the last 14 days before symptom onset; (3) contact with a cluster of confirmed cases or has epidemiological relationship with 2019-nCoV infected cases. •Clinical manifestations: same as the second edition.	Suspected case having any one item of pathogenic evidence stated below:(1) Respiratory tract or blood samples showing positive for real-time PCR test for 2019-nCoV; (2) Respiratory tract or blood samples for viral whole genome sequencing showing high homogeneity to the known novel coronaviruses.	Meets any one of the following criteria: (1) Respiratory distress, respiratory rate (RR) ≥30 breaths/minute, dyspnea, lips slightly cyanosed, (2) Resting state oxygen saturation during inhalation ≤ 93%; (3) The PaO2/ FiO2 ≤ 300 mmHg (1 mmHg = 0.133kPa).
Fifth (outside Hubei)	Combination of any one feature of epidemiological history with two of clinical manifestations to make a comprehensive analysis: •Epidemiological history: (1) a history of travel or residence in Wuhan city and surrounding areas, or other communities where COVID-19 had been reported in the last 14 days before symptom onset; (2) a history of contact with 2019-nCoV infectious cases (with positive nucleic acid test); (3) a history of contact with patients with fever or respiratory symptoms from Wuhan city and surrounding areas, or other communities where COVID-19 had been reported in the last 14 days before symptom onset; (4) contact with a cluster of confirmed cases. •Clinical manifestations: (1) fever and/or respiratory symptoms; (2) imaging features of COVID-19; (3) total white blood cell counts showing normal, decreased, or reduced lymphocyte count in the early onset stage.	Same as the fourth edition.	Same as the fourth edition.
Fifth (in Hubei)	Combination of any one item or no features of epidemiological history in addition to two clinical manifestations to make a comprehensive analysis: •Epidemiological history: same as the fifth edition (outside Hubei Province). •Clinical manifestations: (1) fever and/or respiratory symptoms; (2) with imaging features of COVID-19 (clinically diagnosed case); (3) total white blood cell counts showing normal, decreased, or reduced lymphocyte count in the early onset stage.	Clinically diagnosed case or case having any one item of the following pathogenic evidence:(1) Respiratory tract or blood samples positive for real-time PCR test for 2019-nCoV; (2) Respiratory tract or blood samples for viral whole genome sequencing showing high homogeneity to the known novel coronaviruses.	Same with the fourth edition.
Sixth	Combination of any one feature of epidemiological history with two clinical manifestations to make a comprehensive analysis, or, where there is n clear epidemiological history, needs to show three clinical manifestations: •Epidemiological history: same as the fifth edition (outside Hubei Province). •Clinical manifestations: same as the fifth edition (outside Hubei Province).	Suspected case having any one item of pathogenic evidences as following:(1) Positive real-time PCR test for 2019-nCoV; (2) viral whole genome sequencing showing high homogeneity to the known novel coronaviruses.	Meets any one of the following criteria:(1) Dyspnoea, RR ≥30 breaths/minute; (2) Resting state oxygen saturation during inhalation ≤ 93%; (3) The PaO2/FiO2 ≤ 300 mmHg (1 mmHg = 0.133kPa). •For patients from high attitude areas (over 1,000 meters above sea level) it is necessary to adjust the PaO2/FiO2 using the formula: Pa O2/Fi O2 × [atmospheric pressure (mmHg)/760]. •Manage as severe case if the pulmonary imaging shows leafy lesions or progressive lesions > 50% in 24–48 h.
Seventh	Combination of any one epidemiological history feature in addition to two clinical manifestations to make a comprehensive analysis, and needs to show three clinical manifestations where there is no clear epidemiological history: •Epidemiological history: (1) a history of travel or residence in Wuhan city and surrounding areas, or other communities where COVID-19 had been reported in the last 14 days before symptom onset; (2) a history of contact with 2019-nCoV infectious cases (with positive nucleic acid test); (3) a history of contact with patients with fever or respiratory symptoms from Wuhan city and surrounding areas, or other communities where COVID-19 had been reported in the last 14 days before symptom onset; (4) contact with a cluster of confirmed cases (≥ 2 cases with fever and/or respiratory symptoms occurring within 2 weeks in small areas, such as home, office, class of school, etc). •Clinical manifestations: same as the fifth edition (outside Hubei Province).	Suspected case having any one item of pathogenic or serological evidences as following:(1) positive real-time PCR test for 2019-nCoV; (2) viral whole genome sequencing showing high homogeneity to the known novel coronaviruses; (4) the specific IgM antibody and IgG antibody of 2019-nCoV are reported in serum as positive; or the 2019-nCoV specific IgG antibody in serum changes from negative to positive, or rises in the recovery phase ≥ 4 times above that in the acute phase.	Adult: Same with the sixth edition. Children who meet any one of the following criteria: (1) Dyspnoea (<2 months, RR ≥50 breaths/minute; 1–5 years, RR ≥40 breaths/minute; > 5 years, RR ≥30 breaths/minute), unless affected by fever and crying; (2) Resting state oxygen saturation during inhalation ≤ 92%; (3) Assisted respiration (groan, nasal ala flap, three depression sign), cyanosis, intermittent apnea; (4) lethargy and convulsions; (5) Apastia or feeding difficulties, with dehydration.

* *The “suspected case” was named “observed case” in the first edition*.

### Changes of Case Classification

The criteria for case classification developed considerably over time. In the first edition, three types were described: observed case, confirmed case, and critical case; however, from the second edition onwards, the term “observed case” has been changed to “suspected case,” and the criteria for “severe case” has been added. In the third edition, more clinical manifestations were added, and were classified as moderate, severe, and critical according to the severity of clinical symptoms. In the fifth edition, the form “mild” was added. Hence, in the seventh edition, the types were suspected case or confirmed case, with four clinical levels of severity: mild, moderate, severe, and critical cases.

In the fifth edition, the term “clinically diagnosed cases” was defined for Hubei Province. In Hubei Province, the suspected patients who had imaging features of pneumonia were designated as clinically diagnosed cases. However, this type was merged into “suspected case” in the sixth and seventh editions.

### Changes to “Suspected Case”

The term “observed case” used in the first edition was changed to “suspected case” in the second edition. For a patient to be diagnosed as suspected this had to be based on the epidemiological history and clinical manifestations. From the second to fourth editions, it had to include any two epidemiological history features in addition to clinical manifestations. However, from the fifth to seventh editions, a comprehensive analysis was required which included epidemiological history along with clinical manifestations; cases without clear epidemiological histories were added as a criterion judged by the presence of clinical features.

Epidemiological history initially included direct or indirect contact with related markets in Wuhan, which was mentioned in the first edition. However, this item was deleted from the second edition onwards, but “a history of contact with patients with fever or respiratory symptoms from Wuhan city within the last 14 days before symptom onset, or with a cluster of confirmed cases” was added. Then in the fourth edition, the contact scope was enlarged to include other places where COVID-19 had spread and also included an epidemiological relationship with 2019-nCoV infected cases.

For clinical manifestation, “symptoms without obvious improvement or with progressive severity after 3 days of standardized antimicrobial therapy” was mentioned in the first edition. However, this was deleted from the second edition onwards, and it only included fever, imaging features of COVID-19, and total white blood cell counts showing normal, decreased, or reduced lymphocyte count. In the fifth edition, the fever symptom was expanded and respiratory symptoms added; the criterion of “suspected case” was divided into inside Hubei Province and outside Hubei Province. The suspected patients having imaging features of pneumonia were designated as clinically diagnosed cases.

### Changes in “Confirmed Case”

In the first edition, to define the case as “confirmed,” it was necessary to collect the respiratory tract sample for viral whole genome sequencing, and this needed to show high homogeneity to the known novel coronaviruses. The real-time PCR test for nucleic acid in the respiratory tract or blood samples was added in the second and third editions. The pathogenic detection via blood samples was added in the fourth and fifth editions; the serological evidence was added in the seventh edition.

In the seventh edition, if “the specific IgM antibody and IgG antibody of 2019-nCoV are reported in serum as positive,” or “the 2019-nCoV specific IgG antibody in serum changes from negative to positive, or rises ≥ 4 times in the recovery phase above that in the acute phase,” this is also diagnosed as COVID-19.

### Changes in Clinical Classifications

There was no clear clinical classification in the first three editions. In the first edition, it specified a clear criterion for “critical case,” and the criterion for “severe case” was added in the second and third editions. The clinical stages were added in the fourth edition, which divided confirmed cases into moderate severe and critical stages; the mild stage was then added in the fifth to seventh editions. The mild stage was defined as persons having slight clinical symptoms without any imaging features of pneumonia.

### Changes in “Severe Case”

The criterion for severe case has evolved since the second edition. Compared with this edition, “Oxygen saturation during inhalation ≤ 95%” was modified to “Oxygen saturation during inhalation ≤ 93%” in the third to seventh editions; additionally, the descriptions of rapid Sequential Organ Failure Assessment (qSOFA) score, CURB-65 score, and coalescent pneumothorax were also deleted. The “other combined clinical conditions that necessitate hospitalization” in the second and third editions was also deleted in the latter four editions. The description “pulmonary imaging shows leafy lesions or progressive lesions > 50% in 48 h” was deleted in the fourth and fifth editions but was reincorporated in the sixth and seventh editions. In the sixth edition, “the necessity to adjust the arterial partial pressure of oxygen (PaO_2_)/fraction of inspired oxygen (FiO_2_) for patients from high attitude areas” was added. In the seventh edition, “severe cases” were defined and divided into children and adults.

### Unchanged Criteria

The criteria for critical case were almost unchanged in these seven editions, except that in the third to seventh editions, “respiratory failure” was expanded to “respiratory failure occurs and mechanical ventilation is required,” and “septic shock” was revised to “shows symptoms of shock.”

## Discussion

### Reasons for Changes

We believe there are two major reasons for these changes: our increasing knowledge and awareness of COVID-19 and the feedback from clinical practice.

Because COVID-19 is a new infectious disease, we are still learning its etiology, source of infection, transmission route, capacity for transmission, clinical manifestation, diagnostic criteria, treatment, and other relevant information. In the early stages, the clinical symptoms of COVID-19 are similar to common flu or pneumonia, which only present as fever and/or a cough (3). Hence, only observation and empirical antimicrobial therapy were given for mild cases. When the 2019-nCoV was extracted from patient samples, the antimicrobial therapy was then deleted from the treatment criteria of suspected cases. In response to the rapid increase in the number of cases which were submitted by Wuhan City, many government departments reacted rapidly (6) by establishing a joint prevention and control mechanism, increasing the information about COVID-19, raising public awareness of the outbreak, revising “observed case” to “suspected case,” and providing centralized isolation for suspected cases. Then, with reports of increasing numbers of confirmed and suspected cases in many regions, especially when human-human transmission was confirmed, and many cases were presenting with no history of living in or contact with Wuhan (7), the designation of suspected case was not limited to a person's epidemiological history, and isolated cases whose clinical manifestations met the criteria of suspected case were included.

According to the whole genome sequencing result of 2019-nCoV and compared with other novel coronavirus (8), etiological detection become the main piece of evidence and the core criterion for diagnosis of COVID-19. Relevant research also continued to search for an optimal nucleic acid detection kit for rapid diagnosis, and the rapid real-time PCR test was established (9). When sampling included blood, as well as samples from the respiratory tract, this increased the availability of different specimens (10). Then serological evidence was included in the etiological evidence category based on relevant studies, and this supported bringing the specific antibody positive result into the confirmed criteria.

The frontline doctors and nurses also accumulated more and more clinical experience of seeing and treating COVID-19. Hence, the clinical severity stages were designated according to patients' symptoms from the third edition of the guideline, to facilitate individualized treatment and surveillance of patients. We found some patients' RT-PCR test became positive again after recovery from COVID-19 (11). Moreover, due to the nucleic acid testing being too slow to meet clinical requirements, this resulted in many people not being correctly diagnosed. Hence, in the fifth edition of the guideline, suspected patients who had imaging features of pneumonia in Hubei Province were considered as clinically diagnosed cases, and then given standardized treatment. This “clinically diagnosed case” was canceled when the capacity of nucleic acid detection improved.

We can see that the diagnostic criteria of “severe case” became more detailed and specific. Oxygen saturation during inhalation was reduced from ≤ 95 to ≤ 93%, and qSOFA score, CURB-65 score, and description of coalescent pneumothorax were removed. The qSOFA score and CURB-65 score were usually used to assess the severity and prognosis of community-acquired pneumonia (12), but many studies believed they do not have an ideal correlation with prognosis of COVID-19, the reason being COVID-19 is more dangerous (13). Hence, they were deleted from the diagnostic criteria for severe cases. When confirmed cases came from the high-altitude areas having a low concentration of oxygen, the necessity to adjust PaO_2_/FiO_2_ values for cases from these areas was added in the sixth edition. Also, along with defining suspected and confirmed cases and the accumulation of clinical experiences in children, the “severe cases” definition for children was added in the seventh edition.

### Implications for Diagnostic Criteria Development

Some diagnostic criteria were significantly changed over the seven versions of the guidelines. There were many descriptions added relating to epidemiological histories of suspected cases, this indicates that relevant epidemiological studies should be performed as quickly as possible after an outbreak in order to detect the routes of infection and provide evidence for diagnosis and control. For the diagnostic criteria of COVID-19, from nucleic acid tests to clinical imaging features, from etiological evidence to serological evidence, diagnostic means and methods continue to increase (14). However, we must acknowledge that every diagnostic method has its own strengths and weaknesses. For example, a nucleic acid test may produce false-negative results and has a longer detection time, while imaging tests have a short detection time but accurate results are dependent on the radiologists' skill level. Besides, some diagnostic criteria remain little changed, such as the criteria for severe case which is almost unchanged. This is because when COVID-19 patients enter the critical phase it is often accompanied by organ failure and shock; these changes develop rapidly and have a high fatality rate. Hence, the standard has been maintained for better clinical monitoring and judgment.

When the case definition was gradually broadened as knowledge increased, it had a substantial influence on the proportion of infections being detected as cases. When cases are classified in detail, different types of patients are treated with customized interventions, and medical resources can be properly allocated. A study has found through modeling evaluation that from the first version to the second version, the proportion of infections being detected as cases increased by 7.1 times (95% confidence interval [CI], 4.8–10.9), from the second version to the fourth version it increased by 2.8 times (95% CI, 1.9–4.2), and from the fourth version to the fifth version by 4.2 times (95% CI, 2.6–7.3) (15). After adding the category of “clinically diagnosed cases” in the fifth edition, 13,332 new clinically diagnosed cases were added (16). Many highly suspected patients who did not receive virological testing due to insufficient detection capabilities were able to be isolated in designated hospitals in a timely manner, with priority given to virus detection and treatment, so as to reduce the potential infection rate and mortality. After all clinically suspected cases were able to be tested by the laboratory, the “clinically diagnosed cases” were deleted in the sixth version.

Although the use of radiological evidence to confirm viral pneumonia may be an important alternative to the diagnosis and monitoring of COVID-19, it also brought some problems. This procedure may include some patients with common pneumonia; hence criteria for clinically diagnosed patients also needs to include the nucleic acid results at a later stage to correct the actual number of cases. In addition, with the surge of patients, it was necessary to have enough hospital beds quickly to ensure the early diagnosis and treatment of patients. This requires more funds, equipment, and medical staff input, which poses a challenge to the country's regulatory and economic capabilities (17).

## Conclusions

The nucleic acid test is currently used as a confirmed diagnosis method. In addition, imaging examination and epidemiological history should also be considered as auxiliary diagnosis methods. We suggest approaching COVID-19 diagnosis with caution, doing as much as we can to reduce misdiagnosis and missed diagnoses, exploring and combining different methods, and actively seeking new methods, especially for screening asymptomatic patients and also identifying people who retest as positive again after recovery.

## Data Availability Statement

All datasets generated for this study are included in the article/supplementary material.

## Author Contributions

X-TZ and X-QR: conceptualization. B-HL, TD, and L-LM: writing-original draft preparation. X-TZ, Y-HJ, and X-QR: reviewing and editing. All authors have read and agreed to the published version of the manuscript.

## Conflict of Interest

The authors declare that the research was conducted in the absence of any commercial or financial relationships that could be construed as a potential conflict of interest.
